# Supplementary material for: Ripples in the bottom of the potential energy landscape of metallic glass
Source: Nat Commun. 2024 Feb 14;15:1358. doi: 10.1038/s41467-024-45640-1 (PMC10866862; doi:10.1038/s41467-024-45640-1)
Supplement: Supplementary file 1 — Supplementary Information [file 41467_2024_45640_MOESM1_ESM.docx]

Supplementary Information for

Transition From Low to High Transient Nature of Mechanical Loss in Metallic Glass

Leo Zella^1^, Jaeyun Moon^2#^, Takeshi Egami^1,2,3*^

*^1^Department of Materials Science and Engineering, The University of Tennessee, Knoxville, TN 37996, USA*

*^2^Materials Science and Technology Division, Oak Ridge National Laboratory, Oak Ridge, TN 37831, USA*

*^3^Department of Physics and Astronomy, The University of Tennessee, Knoxville, TN 37996, USA*

*#Present address: School of Mechanical and Aerospace Engineering, Cornell University, Ithaca, NY 14853, USA.*

**Electronic mail:* [*egami@utk.edu*](mailto:egami@utk.edu)

**Supplementary Note 1**

*Linear stress-strain regime*

Simulations at a variety of strains confirm the linear stress-strain regime. Simulations had identical parameters with exception of system size of 4000 atoms.


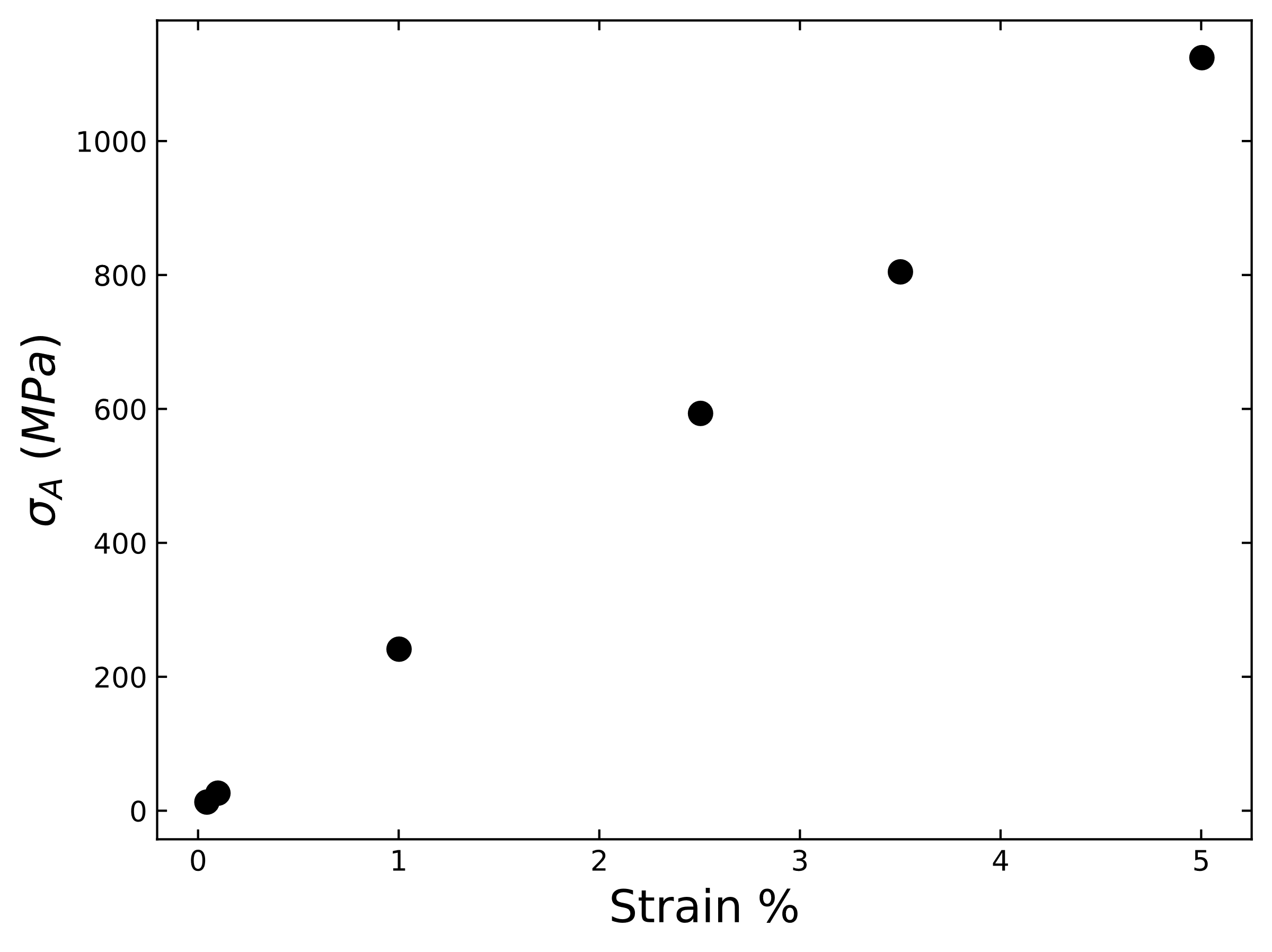


Supplementary Fig. 1. Shear strain magnitude vs. shear stress response. Figure shows the linear stress-strain region.

**Supplementary Note 2**

*Lossy fraction 9 cycles later*

Using the same definition for the lossy fraction of atoms participating in mechanical relaxation as Eq. 2. We calculate lossy fraction 9 cycles later, $\left\langle f_{lossy}(n,n+9) \right\rangle,$to ensure that lossy atoms are not reversible on a longer timescale. The results demonstrate the same trend in Fig. 4. for one cycle later $\left\langle f_{lossy}(n,n+1) \right\rangle.$


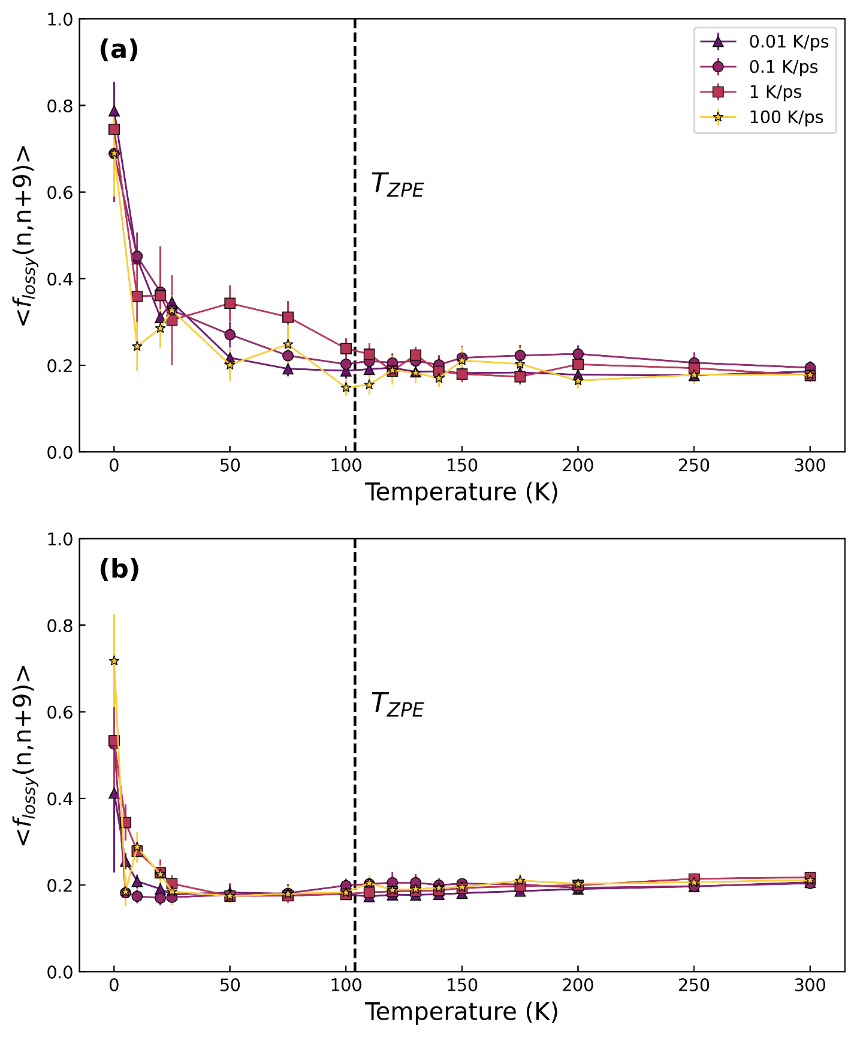


Supplementary Fig. 2. Lossy fraction $\left\langle f_{lossy}(n, n+m) \right\rangle$ for various cooling rates using $m=9$ (nine cycles later) at a strain of (a) 2.5% and (b) 1%. For both comparisons a rapid transition from low transience where lossy atoms mostly remain lossy to high transience is shown with plateau emerging earlier for the 1% results. The dashed line represents the effective zero-point energy temperature which is in the region of high transience. Data is averaged from 20 cycles with error bars representing the standard deviation.

**Supplementary Note 3**

*Average fraction of lossy atoms*

By using the definition of lossy atoms as $\delta_{atom}\in[\frac{\pi}{4},\frac{3\pi}{4}]$ we calculation the average fraction of lossy atoms, $<f_{lossy}>$, as a function of both temperature and cooling rate. We see a weak temperature dependence and a small increase in the fraction with faster cooling rates. These results demonstrate that faster cooling rates produce more regions where these small rearrangements may occur.


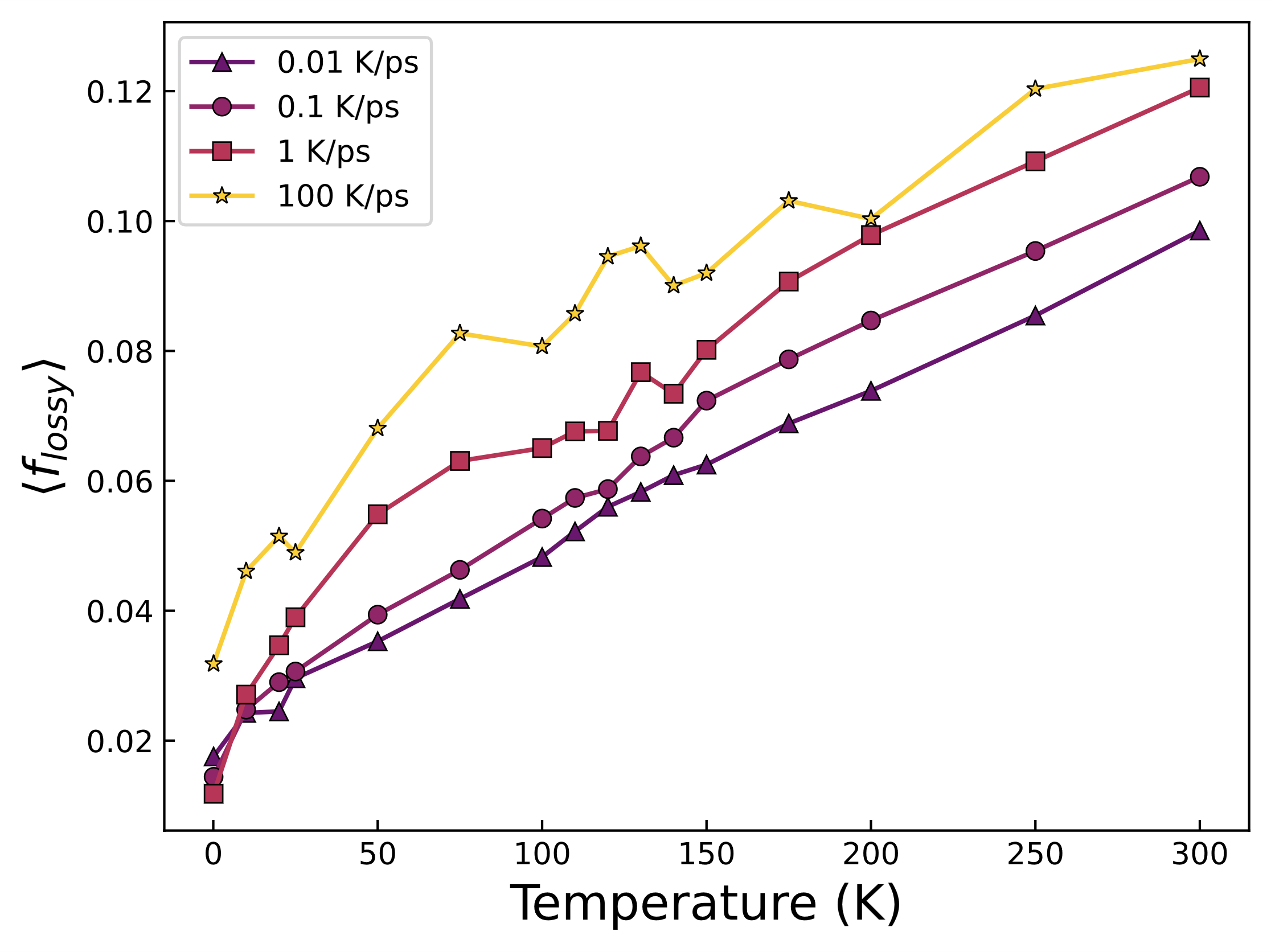


Supplementary Fig. 3. Fraction of lossy atoms vs. temperature for various cooling rates. There is a weak temperature and cooling rate dependence.

**Supplementary Note 4**

*Annealing due to cyclic shear strain*

To understand the role of mechanical annealing, change in potential energy of the system is plotted for the 30 cycles of equilibration/training and the following 30 cycles where data were taken to characterize transience. This is plotted for $0.1 K$ and $300 K$ in Supplementary Fig. 4. Annealing happens rapidly except for the fastest cooled system of $100 K/ps$. Data have been block averaged over a period $\Delta t=100 ps$.


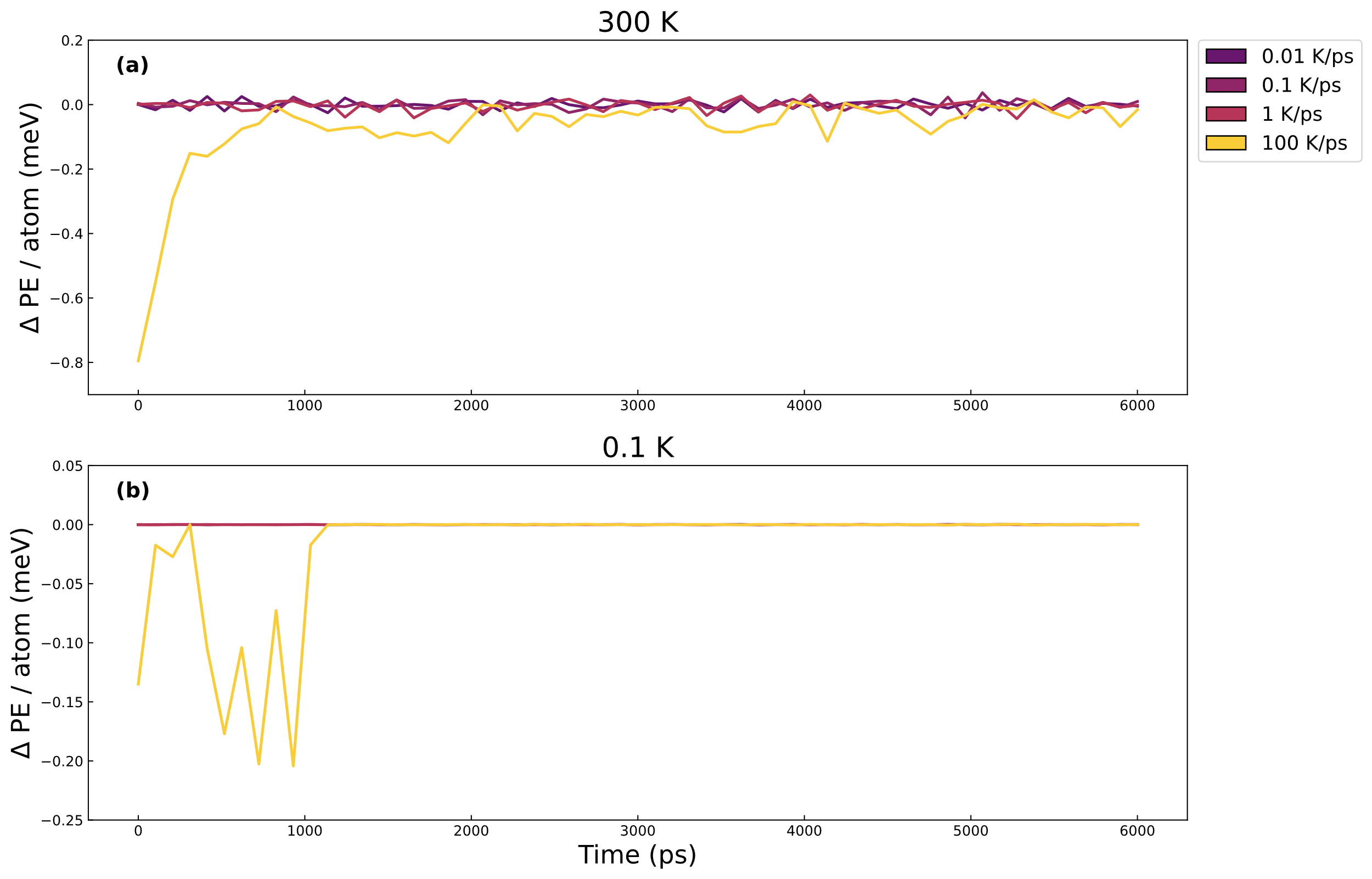


Supplementary Fig. 4. Potential energy of system under mechanical straining for 60 cycles (6000 ps) of sinusoidal straining. First, 30 cycles are the training/equilibration cycles, and the last 30 cycles are for the data collection. Average potential energy change per atom for all four cooling rates at (a) 300 K and (b) 0.1 K

**Supplementary Note 5**

*Sensitivity of transience transition due to lossy atom definition*

To ensure that conclusions are not significantly modified by the width of bounds that define lossy atoms as $\delta_{\mathrm{atom}}\in[\frac{\pi}{4},\frac{3\pi}{4}]$, $\left\langle f_{lossy}(n,n+1) \right\rangle$ is calculated for a variety of bounds for the slowest cooled simulation of $0.01 K/ps$. The bounds we have used for this work are indicated by the solid line and it is shown there is no significant difference in the temperature scaling of transition from low to high transience.


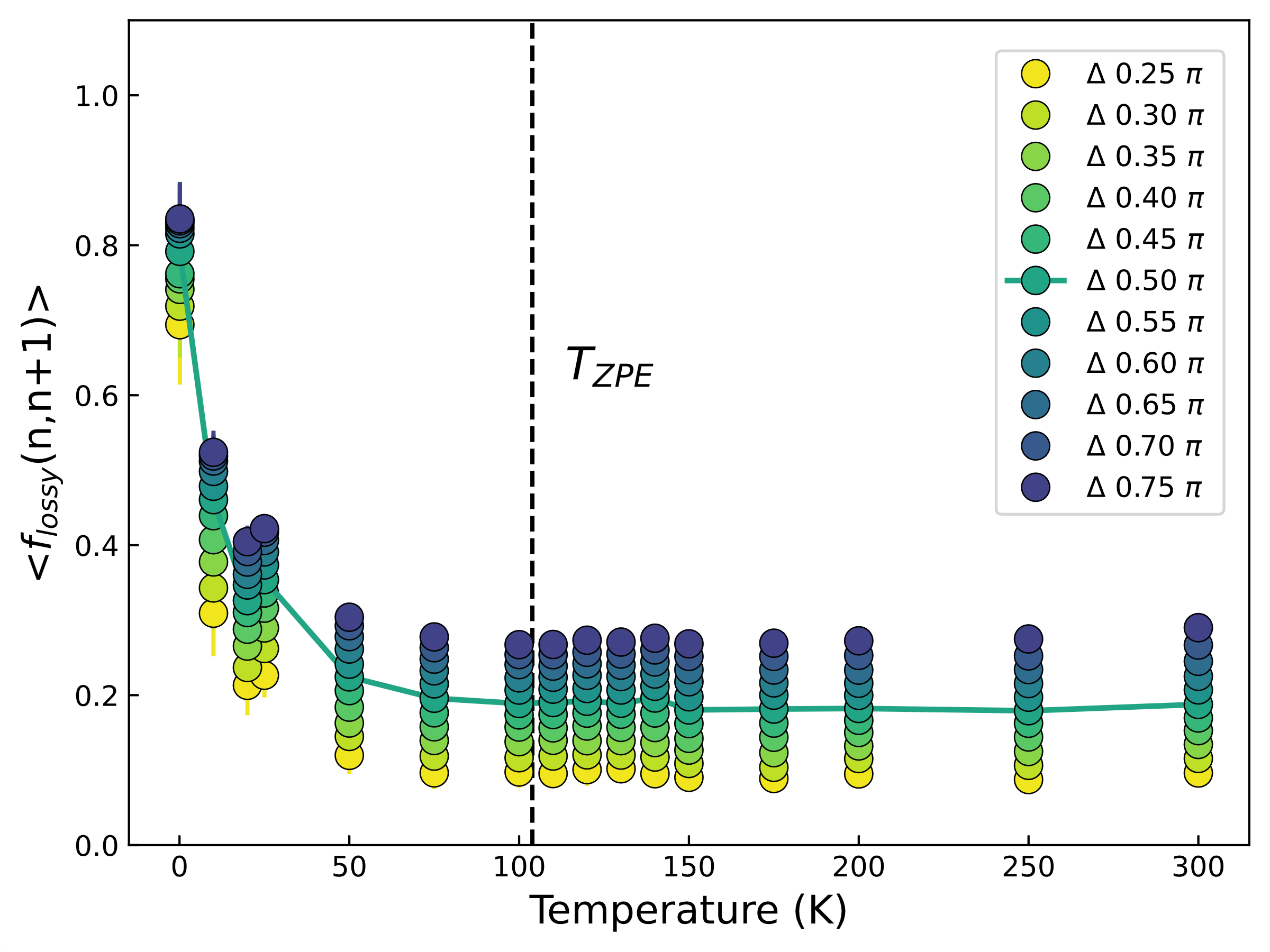


Supplementary Fig. 5. Cycle-to-cycle transience for $0.01 K/ps$ with a variety of widths for the bounds that define lossy atoms. Solid line indicates the definition chosen for our work.

**Supplementary Note 6**

*Vibrational density of states*

Vibrational density of states is calculated according to Eq. 3 in the main text. Results are averaged from 5 different simulations at 0.1 K with different starting velocities.


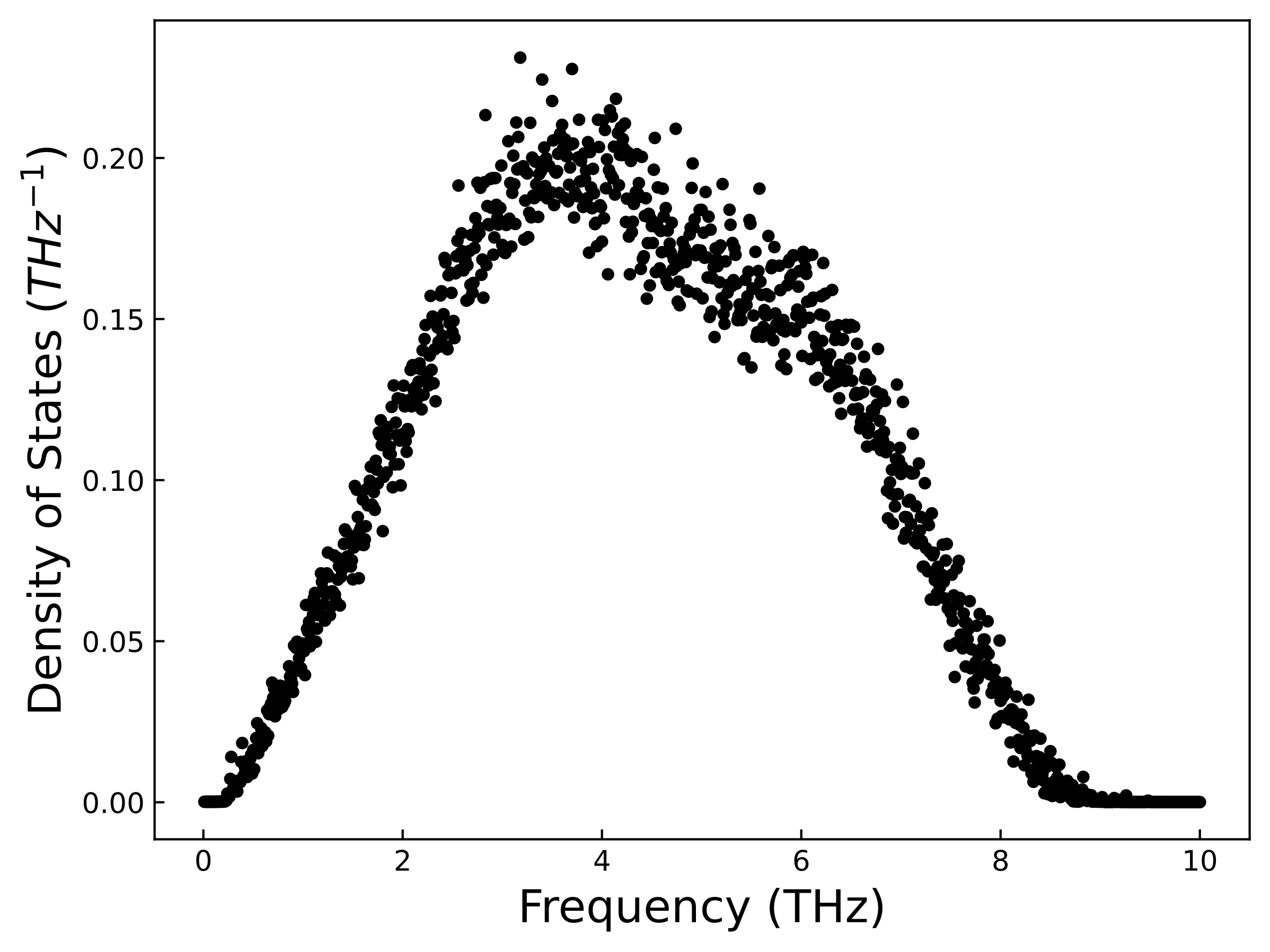


Supplementary Fig. 6. Averaged vDOS from 5 different velocity trajectories with area normalized to 1.
